# Dietary inflammatory index is associated with pain intensity and some components of quality of life in patients with knee osteoarthritis

**DOI:** 10.1186/s13104-020-05277-x

**Published:** 2020-09-21

**Authors:** Vahideh Toopchizadeh, Neda Dolatkhah, Dawood Aghamohammadi, Mahrokh Rasouli, Maryam Hashemian

**Affiliations:** 1 Physical Medicine and Rehabilitation Research Center, Aging Research Institute, Tabriz University of Medical Science, Azadi Ave., Tabriz, Iran; 2 Palliative Care Medicine Department, Faculty of Medicine, Tabriz University of Medical Sciences, Tabriz, Iran; 3 Faculty of Medicine, Tabriz University of Medical Sciences, Tabriz, Iran; 4 Department of Biology, School of Arts and Sciences, Utica College, Utica, USA

**Keywords:** Dietary Inflammatory Index, Osteoarthritis, Pain, Functional status, Quality of life

## Abstract

**Objectives:**

We aim to measure dietary inflammatory index (DII) and its association with functional status, pain intensity and quality of life (QOL) in patients with knee osteoarthritis (KOA). Dietary information from 220 qualified patients with KOA was collected by a 168-item food frequency questionnaire. The functional status, pain intensity and QOL were evaluated by Western Ontario and McMaster Universities Osteoarthritis Index (WOMAC) questionnaire, Visual Analogue Scale (VAS) and SF36 questionnaire respectively.

**Results:**

Linear regression analysis showed that the DII was significantly associated with VAS (p = 0.040; β = 0.151), and physical function (p = 0.039; β = − 0.184), emotional wellbeing (p = 0.048; β = − 0.158) and pain (p = 0.020; β = 0.161) scales and physical health (p = 0.047; β = 0.110) subscale of QOL after adjusting for age, sex, body mass index, and physical activity. There was no significant differences concerning WOMAC across the DII tertiles with and without adjustment to probable confounders (P_trend_ = 0.091 and 0.181, respectively). After adjustment, a significantly increased severe pain odds was observed in the highest tertile of DII score in comparison with the lowest tertile (OR tertile _3 vs. 1_ = 1.55, 95% CI 1.04–2.31; P_trend_ = 0.04).

## Introduction

Knee osteoarthritis (KOA) is a musculoskeletal disorder of an inflammatory nature that affects people around the world, causing pain, physical disability, and lowering the quality of life (QOL) [1]. Previous studies have shown that inflammation play a role in the pathogenesis and the development of osteoarthritis (OA) [2] and an important factor associated with the loss of cartilage and symptoms of the disease [3].

Various studies have shown that one’s dietary habits play an important role in regulating chronic inflammation [4, 5] and demonstrate a “dietary basis” for the inflammatory presentation in rheumatic and musculoskeletal diseases, recommending an organized multidisciplinary approach to manage these conditions [6, 7]. An ideal nutritional pattern is essential for regulating inflammation and oxidative stress, which are interrelated with the immune system [8]. The central conception of the relationship between dietary components and inflammatory and oxidative stress procedures has been well-recognized and corroborated, for instance, in the creation of the dietary inflammation index (DII). DII is a scoring system for measuring the potential of diet in inducing inflammation. DII validity regarding inflammatory markers such as C-reactive protein (CRP) and interleukin 6 has been shown in previous studies [9, 10]. This indicates the capability of this indicator in determining the relationship between inflammatory compliance of diet and chronic disease [11, 12].

Considering the importance of the topic and rarity of studies in this area, especially in patients with KOA, this study was designed to firstly identify the DII in patients with KOA, and secondly, the association of DII with functional status and pain intensity and QOL in these patients.

## Main text

### Procedure

This descriptive cross-sectional study was conducted on 220 individuals with KOA during the March-September 2018. Participants were selected via proportional random sampling from the health centers of Tabriz City and then phoned, a summary explanation of the objectives and process of the research were described and, if they agreed to contribute in the study, they were requested to be present at the selected time in the center.

### Inclusion criteria

Diagnosis of primary KOA defined by the American College of Rheumatology classification criteria [13], age 45 years old and over and no other inflammatory disease such as rheumatoid arthritis.

### Exclusion criteria

Follow a special dietary pattern for medical or other reasons, subjects with neuropathy and sensory impairment, body mass index (BMI) ≥ 35, history of knee joint injection in the last 6 months or the use of corticosteroid medications for a recent month, people who are unable to cooperate in completing questionnaires and procedures for any reason.

### Assessment of dietary intake

A validated 168 items semi-quantitative food frequency questionnaire (FFQ) [14] was used for examining participants’ dietary intakes in previous 1 year. Each food and drink was coded and entered in Nutritionist IV software to assess the average daily intake of energy and nutrients including protein, carbohydrate, fat, saturated fats, monounsaturated fatty acids, polyunsaturated fatty acids, omega-3 fatty acids, and omega-6 fatty acids, cholesterol, fiber, Vitamin A, Vitamin C, Vitamin D, Vitamin E, Vitamin B12, Vitamin B1, Vitamin B2, Vitamin B6, niacin, folate, beta-carotene, iron minerals, zinc, magnesium.

### Dietary Inflammatory Index (DII)

Dietary intakes of 29 abovementioned food parameters were used to calculate the DII. In order to reduce between-subject variation, dietary intake was adjusted to the total daily energy intake using residual method [15]. The food information then was linked to the representative regional database, which estimates the global average intake of each nutrient with its standard deviation [16]. To calculate the subject’s exposure relative to the standard global mean, the standardized meanings were subtracted from these values and divided into the corresponding standard deviations. The z-scores obtained from this method turned into percentiles and doubled, and then “1” declined. Finally, the centralized percentiles for each parameter were multiplied by their related total inflammatory effect score and the total obtained values were added together to obtain an overall DII. DII = b1*n1 + b2*n2…..b29*n29, where b is the literature-derived inflammatory effects score for each food parameter and n is the food parameter-related centered percentiles, which were achieved from the participants’ dietary data. Additionally, we created groups based on the DII tertiles.

### Demographics characteristics

For all participants, the demographic information was gathered via a general information questionnaire. Body weight and height were measured by digital scale (Seca, Hamburg, Germany) and a non-stretched tape measure (Seca, Hamburg, Germany), respectively. The Persian version of International Physical Activity Questionnaire (IPAQ) was applied to measure physical activity [17, 18].

### Visual analogue scale (VAS)

VAS (0–10 cm pain scale: 0 = none and 10 = intolerable) was applied to measure the pain intensity in the participants. The validity of using this scale for assessing the pain intensity has been demonstrated in the studies [19].

### The Western Ontario and McMaster (WOMAC) index

WOMAC index was applied to measure the functional status of participants by considering twenty-four items categorized into three subscales: pain (5 items), stiffness (2 items) and physical function (17 items) [20]. It is one of the frequently used tools to quantity the physical performance related with OA [21]. The WOMAC scores can be linearly changed to a 0–96 scale, with higher scores representing more severe symptoms and impaired function. The validity and reliability of the Persian version of WOMAC index have been established in studies [22].

### 36-item short form health survey (SF-36)

The Persian version SF36 was used to measure QOL in participants. The questionnaire has 36 questions, which assesses eight different areas of health with respect to the physical and emotional aspects. Likert scales and yes/no options are applied to evaluate function and well-being. Scales are standardized with a scoring process to achieve a total score fluctuating from zero to 100. Its validity and reliability have been established in Iran [23].

### Statistical analysis

The 168-item semi-quantitative FFQ, categorizes the food items into twenty-two groups. It has been recommended that the sample size for such studies to be considered as ten subjects per variable [24]. Consequently, the sample size was calculated as 220 subjects.

Normal distribution of variables was tested using Kolmogorov–Smirnov test. Linear regression and Chi square tests were applied to examine the trend of continuous and categorical variables across DII tertiles, respectively. The relationships between the DII score and pain intensity, functional disability and QOL were analyzed by linear regression analysis (Model 0, unadjusted and Model I, adjusting for age, sex, physical activity and BMI. Odds ratio (ORs) and 95% confidence interval (CI) of severe pain and disability across DII tertiles were measured by logistic regression analysis, whereas the lowest tertile of DII considered as the reference. The SPSS version 17.0 (IBM SPSS Statistics for Windows, Version 17.0. Armonk, NY: IBM Corp) has been used to analyze data.

## Results

From 348 subjects who were screened, a total of 220 patients with KOA were qualified for inclusion in the investigation. Demographic characteristics of the participants according to the DII tertiles are shown in Table 1. There was a significant difference concerning the gender of patients across the DII tertiles. Most of men (32 out of 68) were in the top tertile of the DII, while most of women (56 out of 152) were in the first tertile of the DII (p = 0.033). Additionally, subjects in top tertile had significantly higher BMI than two other tertiles (p = 0.032). There was no significant difference in other demographic variables across tertiles of DII.

**Table 1:** General characteristics of patients with knee osteoarthritis across the DII tertiles

Variable	DII score						p value*
	DII Score Tertile 1 (< − 1.3) (n = 73)		DII Score Tertile 2 (− 1.3 to 4.5) (n = 73)		DII Score Tertile 3 (> 4.5) (n = 74)		
Age (years)	0.94* ± 57.85		1.17* ± 54.92		1.21* ± 57.91		0.091
BMI	3.48* ± 36.41		0.54* ± 29.65		0.69* ± 29.66		0.032
Sex							
Male	17	25.00	19	27.94	32	47.06	0.033
Female	56	36.84	54	35.53	42	27.63	
Education							
Illiterate	32	26.91	35	32.71	40	37.38	0.173
Low literate and Diploma	31	43.66	22	30.99	18	25.35	
College	10	23.81	16	38.10	16	38.10	
Occupation							
Unemployed	34	29.57	39	23.91	42	36.52	0.477
Employed	23	32.86	25	35.71	22	31.43	
Retired	16	45.71	9	25.71	10	28.57	
Physical activity							
Low	48	65.75	39	53.42	40	54.05	0.398
Middle	20	27.39	21	28.76	18	24.32	
High	5	6.84	13	17.80	16	21.62	

Data are presented as mean ± SD or n (%)

Chi square test was applied for categorical variables; Linear regression was applied for continuous variables

*BMI* body mass index, *DII* dietary inflammatory index, *yrs* years

*p-value < 0.005 were deemed significant

Data on the pain intensity, functional status and scales and subscales of QOL in the patients across the DII tertiles are shown in Table 2. The finding of linear regression after adjusting for age, sex, BMI and physical activity showed that here were significant differences concerning pain intensity (VAS) and physical function (PF), role limitation due to physical health (RP), social function (SF) and pain (P) scales and physical health (PH) subscales of QOL across the DII tertiles (p for trend of 0.012, 0.014, 0.010, 0.049, 0.043 and 0.045 respectively). According to the adjusted standardized regression coefficients, higher DII scores were associated with higher pain intensity (β = 0.171) and lower PF (β = − 0.184), emotional well-being (EW) (β = − 0.158) and P (β = − 0.161) scales and PH (β = − 0.142) subscales of QOL (Additional file 1).

**Table 2:** The differences on functional status, pain intensity and quality of life among DII score tertiles of patients with knee osteoarthritis

Variable	DII score			p for trend^a^	p for trend^b^
	DII Score Tertile 1 (< − 1.3) (n = 73)	DII Score Tertile 2 (− 1.3 to 4.5) (n = 73)	DII Score Tertile 3 (> 4.5) (n = 74)		
VAS	0.20 ± 4	0.01 ± 6	0.03 ± 6	0.001	0.012
WOMAC	2.21 ± 40	2.32 ± 44	2.12 ± 45	0.181	0.091
QOL					
PF	3.80 ± 55.54	3.95 ± 47.40	3.71 ± 41.23	0.031	0.014
RP	57.43 ± 2.88	3.31 ± 50.51	41.61 ± 3.73	0.004	0.010
RE	3.73 ± 35.14	3.88 ± 38.36	3.78 ± 30.59	0.351	0.215
E/F	2.27 ± 50.34	2.33 ± 49.38	2.1 ± 44.18	0.111	0.085
EW	1.92 ± 44.99	2.07 ± 48.83	1.70 ± 44.43	0.207	0.102
SF	2.53 ± 55.91	2.65 ± 53.42	2.4 ± 47.60	0.061	0.049
P	2.67 ± 64.73	2.69 ± 58.60	2.6 ± 54.52	0.025	0.043
GH	2.5 ± 50.55	2.91 ± 47.47	2.97 ± 48.59	0.737	0.126
PH	228.29 ± 7.58	204.04 ± 5.02	186.98 ± 4.18	0.011	0.045
MH	186.47 ± 3.79	190.03 ± 8.61	166.82 ± 3.51	0.517	0.147

*DII* dietary inflammatory index, *EF* energy/fatigue, *EW* emotional well-being, *GH* general health, *MH* mental health, *P* pain, *PF* physical function, *PH* physical health, *QOL* quality of life, *RE* role limitation due to emotional problems, *RP* role limitation due to physical health, *SF* social function, *VAS*, visual analogue scale, *WOMAC*,Western Ontario and McMaster Index

^a^Linear regression across DII score tertiles

^b^Linear regression across DII score tertiles with adjustment for age, sex, body mass index and physical activity

Crude and adjusted odds ratios and 95% confidence intervals for the association between DII and severe pain and disability according to tertiles of DII score in patients with KOA are presented as Table 3. After adjusting for some potential confounders, a significantly increased severe pain odds was observed in the highest tertile of DII score in comparison with the lowest tertile (OR tertile 3 vs. 1 = 1.55, 95% CI 1.04–2.31; P trend = 0.04).

**Table 3:** Odds ratios and 95% confidence intervals for the association between DII and severe pain and disability according to tertiles of DII score in patients with knee osteoarthritis

	DII score			p for trend^a^	Continuous OR	95% CI
	DII Score Tertile 1 (< − 1.3) (n = 73)	DII Score Tertile 2 (− 1.3 to 4.5) (n = 73)	DII Score Tertile 3 (> 4.5) (n = 74)			
VAS						
Model 0	Ref.	1.19 (0.66–1.75)	1.33 (0.88–2.05)	0.17	1.22	(0.71–1.91)
Model I	Ref.	1.05 (0.72–1.87)	1.55 (1.04–2.31)	0.04	1.48	(1.26–2.02)
WOMAC						
Model 0	Ref.	1.09 (0.59–1.46)	1.59 (0.77–1.99)	0.78	1.40	(0.91–1.61)
Model I	Ref.	1.18 (0.48–1.84)	1.63 (0.61–2.13)	0.09	1.52	(0.58–1.90)

Model 0, Crude; Model I, Adjusted for age, sex, body mass index and physical activity

VAS was converted to tertiles, considering the first tertile (VAS < 4 cm) as mild, second tertile (4 cm ≤ VAS ≤ 7 cm) as moderate, and the last tertile (VAS > 7 cm) as severe [40]. WOMAC was converted to tertiles, considering the first tertile (WOMAC < 24 points) and second tertile (25 cm ≤ WOMAC ≤ 48 cm), and the last tertile (WOMAC > 48 cm)

*DII* dietary inflammatory index, *VAS* visual analogue scale, *WOMAC* Western Ontario and McMaster Index

^a^Logistic regression analysis

## Discussion

The present study found that consuming a diet with higher DII score was related with higher pain intensity and lower QOL. After adjusting for potential covariates, KOA patients with the highest DII score had significantly higher VAS, and lower QOL in terms of PF, RP, SF and P scales and PH subscale. In this study, we observed that higher DII of diet was associated with 48% higher likelihood of having severe pain in patients with KOA. However DII score was not significantly associated with functional status measured by WOMAC the patients. These findings reinforce the rising body of evidences on the promising role of foods with lower inflammatory capacity in decreasing the odds of severe symptoms of KOA.

The effect of diet on the inflammatory markers such as CRP has been well recognized in numerous observational investigations, with utmost investigations indicating that healthier diets may be related with lesser inflammation [25–28]. In recent years, the role of systemic low-grade inflammation in the pathogenesis of OA has been well-recognized [29]. In patients with OA, upper systemic concentrations of pro-inflammatory cytokines are linked with further pain and dysfunction [30]. Distribution of these cytokines from the synovial fluid into the cartilage tissue plays a key role in cartilage matrix defect by arousing chondrocyte catabolic pathway and obstructing anabolic pathway [31]. Chronic low-grade inflammation may be a main stimulus of continuing joint degeneracy, therefore disturbing cartilages and bones structures characteristic for KOA. Additionally, inflammatory cytokines can induce muscle dysfunction and decrease the pain threshold [32].

Studies investigating the relationship between DII and OA are rare. Along with the results of this study, in A large cross-sectional study, Veronese et al. [33] showed that higher DII is linearly associated to increased prevalence of radiographic symptomatic KOA. Subjects with the highest DII score had a significantly increased occurrence of radiographic symptomatic KOA by almost 40%. Furthermore, it has been found that extra-cellular matrix alterations and breakdown products may increase the inflammation and cartilage damage [34]. So it is probable these alterations play a further role in the association between DII and KOA. A pro-inflammatory dietary pattern can further stimulate these processes. It has also been shown that the DII has a reverse relationship with healthy dietary patterns (such as the Mediterranean diet) that can protect against KOA [35].

To our knowledge, our study is the first that examined the relationship between DII score and scales and subscales of QOL. However, the association of systemic inflammation with high levels of inflammatory and pro-inflammatory cytokines with reduced QOL in general and specific components including pain, physical and emotional health have been shown in previous studies [36–39].

## Conclusion

In conclusion, DII score was associated with pain intensity (VAS) and PF, RP, and P scales and PH subscales of QOL in patients with KOA with and without adjustment for age, sex, BMI and physical activity. Higher DII in the diets was related with higher odds of severe pain in patients with KOA.

### Limitation

First, we can’t conclude confident cause and effect associations because of the cross-sectional design of the study. Relatively small sample size in second limitation. Third, the possibility of recall bias should be considered. Fourth, in our study, data are available on 29 of the 45 food parameters; lack of data on the residual food parameters intake is a limitation. Fifth, self-reported dietary approaches such as FFQ are exposed to bias, the incapability to quantity this bias and adjust for it is a limitation.

## Supplementary information

**Additional file 1.** Standardized regression coefficients (B) and their standard error (SE) and *p* value of the association between DII score and pain intensity, functional status and quality of life in patients with knee osteoarthritis.

## Data Availability

All the necessary data are presented herewith. However if needed, raw data on excel format can be availed on reasonable request from the corresponding author.

## Abbreviations

BMI: body mass index
CI: confidence interval
DII: dietary inflammatory index
EW: emotional wellbeing
EF: energy/fatigue
FFQ: food frequency questionnaire
GH: general health
KOA: knee osteoarthritis
OA: osteoarthritis
P: pain
PF: physical function
QOL: quality of life
RE: role limitation due to emotional problems
RP: role limitation due to physical health
SF: social function
SD: standard deviation
VAS: visual analogue scale
WOMAC: Western Ontario and McMaster Universities Osteoarthritis Index
