# Supplementary material for: Dietary inflammatory index is associated with pain intensity and some components of quality of life in patients with knee osteoarthritis
Source: BMC Res Notes. 2020 Sep 21;13:448. doi: 10.1186/s13104-020-05277-x (PMC7507718; doi:10.1186/s13104-020-05277-x)
Supplement: Supplementary file 1 — Additional file 1. Standardized regression coefficients (B) and their standard error (SE) and p value of the association between DII score and pain intensity, functional status and quality of life in patients with knee osteoarthritis. [file 13104_2020_5277_MOESM1_ESM.doc]

Additional file 1

Standardized regression coefficients (B) and their standard error (SE) and p-value of the association between DII score and pain intensity, functional status and quality of life in patients with knee osteoarthritis

| Variables | | Model 0^a^ | | | Model 1^b^ | | |
| --- | --- | --- | --- | --- | --- | --- | --- |
|  |  | β^a^ | SE^a^ | p-value^a^ | Β^b^ | SE^b^ | p-value^b^ |
| VAS | | 0.185 | 0.032 | 0.002 | 0.171 | 0.043 | 0.011 |
| WOMAC | | 0.091 | 0.132 | 0.091 | 0.042 | 0.154 | 0.319 |
| QOL | PF | -0.095 | 0.064 | 0.130 | -0.184 | 0.025 | 0.020 |
|  | RF | 0.085 | 0.034 | 0.267 | 0.107 | 0.037 | 0.101 |
|  | RE | 0.012 | 0.078 | 0.894 | 0.038 | 0.032 | 0.808 |
|  | E/F | 0.036 | 0.015 | 0.859 | 0.028 | 0.042 | 0.850 |
|  | EW | -0.120 | 0.071 | 0.070 | -0.158 | 0.029 | 0.039 |
|  | SF | -0.076 | 0.081 | 0.512 | -0.045 | 0.064 | 0.748 |
|  | P | -0.110 | 0.028 | 0.098 | -0.161 | 0.043 | 0.020 |
|  | GH | -0.074 | 0.068 | 0.450 | -0.081 | 0.045 | 0.310 |
|  | PH | -0.020 | 0.057 | 0.094 | -0.142 | 0.068 | 0.045 |
|  | MH | -0.088 | 0.072 | 0.249 | -0.108 | 0.219 | 0.101 |

^a^ Model 0, Crude; ^b^ Model I, Adjusted for age, sex, body mass index and physical activity

EF: energy/fatigue, EW: emotional well-being, GH: general health, MH: mental health, P: pain, PF: physical function, PH: physical health, QOL: quality of life, RE: Role limitation due to emotional problems, RP: Role limitation due to physical health, SF: social function, VAS, visual analogue scale, WOMAC, Western Ontario and McMaster Index
